# The urothelial barrier in interstitial cystitis/bladder pain syndrome: its form and function, an overview of preclinical models

**DOI:** 10.1097/MOU.0000000000001147

**Published:** 2023-11-20

**Authors:** Charlotte van Ginkel, Robert E. Hurst, Dick Janssen

**Affiliations:** a Department of Urology, Radboud university medical Center, Nijmegen, The Netherlands; b Glycologix, Inc., Boston, Massachusetts, USA

**Keywords:** bladder pain syndrome/ interstitial cystitis, in vitro studies, in vivo studies, urothelial barrier

## Abstract

**Purpose of review:**

Investigating bladder pain syndrome/interstitial cystitis (IC/BPS) preclinically is challenging. Various research models have been used to mimic the urothelial barrier closely and replicate the disease. The aim of this review is to discuss preclinical research related to the urothelial barrier in context of IC/BPS.

**Recent findings:**

In vivo models mimic IC/BPS mainly with toxic substances in the urine, with protaminesulfate and proteoglycan deglycolysation resembling a temporary impaired barrier as seen in IC/BPS. This temporary increased permeability has also been found in vitro models. Glycosaminoglycan replenishment therapy has been described, in vivo and in vitro, to protect and enhance recover properties of the urothelium. The roles of immune and neurogenic factors in the pathogenesis of IC/BPS remains relatively understudied.

**Summary:**

Preclinical studies provide opportunities to identify the involvement of specific pathologic pathways in IC/BPS. For further research is warranted to elucidate the primary or secondary role of permeability, together with inflammatory and neurogenic causes of the disease.

## INTRODUCTION

Bladder pain syndrome/interstitial cystitis (IC/BPS) is a well known nonbacterial inflammatory condition of the bladder. The causes of IC/BPS remain speculative, but there are similarities with other inflammatory bladder conditions, such as eosinophilic cystitis, ketamine induced cystitis and post radiation cystitis [1–3]. IC/BPS and these conditions share chronic inflammatory changes in the lamina propria, including acute hemorrhage, immune cell invasion, erosion, granulation tissue formation and abnormal vascularization. They also present similar clinical symptoms as well, such as hematuria, pain, urgency, and frequency [1,2,4]. In all these diseases, the urothelium and its barrier structures are compromised and underlaying layers are inflamed [2,3,5,6]. The loss of the urothelial barrier is considered a key factor in the development of IC/BPS.

Multiple in vivo and in vitro research models have been used to explore the above-mentioned theory [7–11,12^▪^,^13^,^14^^▪▪^,^15^,^16^]. However, investigating IC/BPS preclinically poses challenges due to the unclear cause of the disease, leading to unfocussed and sometimes conflicting research findings [17,18]. Additionally, various research models have been used to mimic the urothelial barrier closely and replicate the disease, further complicating the research landscape. Understanding the composition and function of the urothelium is crucial for selecting appropriate research models for IC/BPS.

In response to these difficulties, this article aims to review preclinical research related to the urothelial barrier in the context of IC/BPS, focusing on cell biology, particularly the glycosaminoglycan-layer (GAG-layer), and various in vivo and in vitro models. 

**Box 1:**
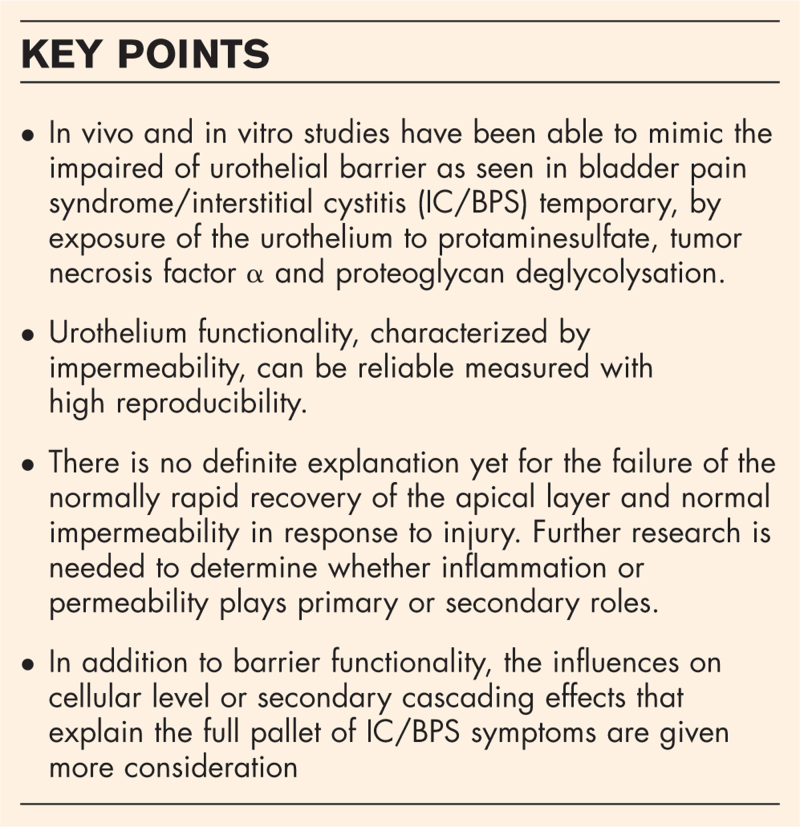
no caption available

## FUNCTIONAL ANATOMY OF THE UROTHELIUM

To understand the pathophysiological changes that occur in IC/BPS, the functional anatomy of the urothelium is important. The bladder urothelium consists of three layers: the basal-, intermediate- and apical layer [19]. In studying IC/BPS the focus is on the apical layer, composed of umbrella cells, which acts as a first barrier against bacteria and toxic solutes in the urine. These umbrella cells, characterized by their rounded shape, are terminally differentiated cells, often multinucleate and formed through fusion of differentiating intermediate layer cells. They possess a cytoskeletal network that is connected to abundant cytoplasmic vesicles with cell membrane components that are able to fuse with the apical membrane, allowing them to resist stretch during bladder filling and emptying [20].

Together with different surface molecules, umbrella cells form an exceptionally tight intercellular barrier. This is mostly mediated through interconnected tight junction networks, which are formed by occludens, claudins, zonula occludens 1 (ZO-1) and junctional adhesion molecule 1 (JAM-1) proteins. Additionally, an asymmetric unit membrane (AUM) composed of uroplakin subunits, covers the umbrella cells. Uroplakins have four subtypes: UPIa, UPIb, UPII and UPIII, with specific binding patterns [20]. Mucins seem to contribute in the hydrophilic surface as antiadherence molecule. The role of lectins remains unclear to date. The GAG layer acts as another highly impermeable hydrophilic barrier covering the apical cell surface of the bladder [21,22]. The loss of the urothelial barrier, specifically the GAG layer, is considered important in the pathophysiology of IC/BPS.

## GLYCOSAMINOGLYCANS AND PROTEOGLYCANS

GAGs are polymers composed of sulfated or unsulfated disaccharide units, including chondroitin sulfates (CS), dermatan sulfates (DS), heparan sulfates (HS) and hyaluronan (HA) [23]. Other GAG's such as keratin sulfate or heparin go beyond the scope of this review. The distribution of disaccharides in GAGs is not uniform, suggesting potential biological information carried by their sequence. With the exception of HA, all GAGs are covalently attached to proteins, forming proteoglycans (PGs). PGs range in size from several million molecular weight to smaller proteins with one or two GAG chains [23]. They are commonly found in connective tissues, providing structural support, and on cell surfaces, where they function as receptors or modulators of growth factors [24].

PGs are intracellularly formed in the Golgi system and transported towards the cell membrane [25]. PGs are abundant on bladder urothelium and form an antiadherence barrier against toxic substances in urine. Analysis revealed four main types of PGs, ranging from 84 to 280 kDa (small PGs), the majority of these PGs consists of CS-PGs (29%) and HS-PGs (55%) [26]. The density of the GAG chains on the surface is estimated to be between 5 and 50 chains deep, creating a highly negatively charged environment, effectively excluding ionic substances and resembling a preferentially bound layer of water. This water layer/salt exclusion zone acts as a first line of defense [13,26].

## IN VIVO

Several animal models have been described for studying IC/BPS. Birder *et al.* categorized the animal models broadly into three groups: bladder-centric models, where the circumstances in the bladder are mimicked with toxic substances in urine, such as cyclophosphamide (CYP), protamine sulfate (PS) and fewer used hydrogen chloride (HCl) [24,26,27]. Among in vivo studies 85% utilized this model type [28^▪▪^]; models with complex mechanisms, this involves altering factors outside the bladder, such as manipulating the central nervous system. This is used in 8% of total [28^▪▪^]; psychological and physical stressors models, such as natural occurring models [feline interstitial cystitis (FIC)] or manipulating environmental factors [29,30], compromising 5% of total [28^▪▪^]. Mouse models are used regularly due to their availability and ease of genetic modification. However, rat models are preferable for translation to humans and allow repetitive sampling better [31].

FIC has similarities with IC/BPS, mainly the nonulcer form (ESSIC type 2) [30]. Both conditions exhibit the loss of the urothelium barrier, characterized by urothelial denudation and changes in epithelial proteins such as ZO-1, E-cadherin and uroplakins, and remodeling of the lamina propria [32,33]. These combined affects sensory signaling, resulting in IC/BPS like symptoms: hypersensitivity, pain, and urgency. However, the utility of FIC as model is limited.

CYP induced cystitis is widely used, CYP causes bladder toxicity through its metabolite acrolein, resulting in hemorrhagic cystitis [7]. This massive local inflammation mimics the ulcerative form of IC/BPS (type 3), unlike FIC. In rats, this inflammation is characterized by increased cytokine levels such as interleukin (IL)-1 and IL-4, accompanied by expected behavioral changes. However, there are limitations to this model, as the effect of CYP varies based on gender and strain in rodents [17,34]. And one can argue that is likely more representative of ketamine induced cystitis, compared to true ulcer type IC/BPS.

PS, an arginine-rich protein, increases the permeability of the urothelium [17,35]. Its exact mechanism remains unclear, but clinically, PS is used to counteract the anticoagulant effect of heparin, a small GAG. One hypothesis is that the positive charge neutralizes the negative charge of the GAG layer. Another hypothesis proposes PS to act by forming ion channels in the membrane that kills the apical cells [20]. The balance between these mechanisms may depend upon the concentration of PS, hence low concentrations increased permeability without causing loss of umbrella cells, while higher concentrations resulted in the loss of umbrella cells and subsequent development of underlying intermediate cells into new umbrella cells [36]. These new cells reform a tight layer considering the induction of ZO-1 and uroplakin, which led to recovery of the inflammation after 24 h [17].

In vivo studies involving specific PG deglycosylation have been conducted in rats [8,9]. This model demonstrates clinical and pathophysiological features like IC/BPS. Enzymatic digestion of CS and HS led to a micturition reflex excitability, decreased pain sensory threshold, irregular loss of uroplakins, increased neutrophils and heightened fos immunoreactivity in L6/S5, indicating noxious signaling from the bladder due to GAG digestion [9]. Digestion of CS and DS increased permeability without causing visible damage to the urothelial structure [36]. Another example is the hyaluronidase model, where intravesical HA digestion led to urothelial changes resembling those observed in IC/BPS, such as chronic inflammation, fibrosis, denudation, mast-cell activation and abnormal expression of UPIII and ZO-1 [8].

### Challenges in vivo

Although these models have provided valuable insights into the consequences of compromising the bladder's defenses, there are limitations to these models. Firstly, in vivo models are expensive, require the sacrifice of numerous rodents and are technically challenging to manipulate. Furthermore, they do not replicate the long-term aspects of the disease because of the rapid regeneration of the urothelium within a week. The urothelium, normally quiescent with minimal cell division, becomes one of the fasted dividing tissues in the body after injury, emphasizing the crucial role of an intact urothelial barrier function. The temporarily aspect in treatments for IC/BPS (such as dimethyl sulfoxide or chlorpactin), indicates an unidentified long-term process that counteracts normal regenerative processes and leads to de-differentiation and/or loss of umbrella cells. Some hypothesizes that increased Antiproliferative factor (APF) activity, which has been described in IC/BPS patients, may contribute to this disruption in urothelial proliferation and repair mechanisms [37].

## IN VITRO

In light the limitations of animal models, several in vitro models have been developed to study specific molecular mechanisms. However, it is important to note that clinical and in vivo studies have identified complex interactions between immune and bladder cells, which are not duplicated by current in vitro models. Only few models form a mature urothelium in culture, Lavelle and co-workers use primary cells without the intermediate step of culturing [35]. Janssen *et al.* used a primary porcine urothelial cell (NPU) model from urothelial biopsies obtained from slaughterhouse pig bladders [13].

An early model used HBT-4, a human urothelial cell line derived from bladders with transitional cell carcinoma, but it does not form tight monolayers like normal urothelium [14^▪▪^,^15^]. Another cell line used is H-BLAK, a primary human bladder cell line isolated from normal bladders [38]. Cell lines are considered inferior to primary cell culture models, because of their mostly carcinogenic origins and reduced urothelial differentiation capabilities that are acquired during immortalization and sub-culturing.

Primary human urothelial cells are typically obtained from nontumorous bladder tissue from cystectomies, mainly performed because of malignancies, rising concerns about the concept of healthy and normal urothelial cells [39^▪▪^]. Furthermore, obtaining human bladder urothelial tissue is difficult, but would allow for potential access to IC/BPS tissue. Much easier accessible, porcine urothelial biopsies offer an alternative source that closely resembles human urothelium in various cell properties [12^▪^,^13^]. For example, NPU exhibit similar distributions of GAGs and urothelial barrier markers (umbrella cells, tight junctions, and adherence junctions) as human urothelium [12^▪^,^13^,^40^]. Moreover, when derived from an abattoir, experimental animals do not need to be involved.

Commonly used in vitro models bear resemblance to bladder-centric animal models, as inflammation is induced using toxic substances. For instance, PS and tumor necrosis factor alpha (TNF-α) have been demonstrated to elevate inflammatory cytokines IL- 6 and IL-8. Furthermore, these models also result in a temporary increased permeability [15,38]. Treatment with exogenous GAGs reduces the inflammatory response and protects the functionality of the urothelial barrier [12^▪^,^13^,^15^]. Moreover, HA treatment promotes the expression of sulfated GAGs [15].

## BARRIER MEASUREMENTS

Functionality of the barrier is characterized by impermeability. Firstly, this can be measured with transepithelial electrical resistance (TEER), which assesses the flow of ions across a membrane in response to applied voltage [41]. A high TEER indicates a strong barrier function, as ions carry electrical current [41], whereas a low TEER indicates greater permeability. The urothelium typically has a TEER of approximately 3000 Ω cm^2^, whereas other epithelial types usually exhibit TEER values of 1000 Ω cm^2^ or lower. This method is sensitive and dependable for monitoring permeability over time, the low voltage applied does not cause cell damage [13].

Secondly, another method measures diffusion of neutral different sizes of molecules and/or ions, for example urea, isotope-labeled water FITC-d or radioactive ^89^Rb^+^. Difficulties with these methods are the high variability in permeability depending on molecular size and interaction with cell membranes. TEER and diffusion methods may yield different results due to their measurements of different flows, respectively transcellular versus the combination of trans- and paracellular [41,42].

Moreover, Towner *et al.* recently demonstrated the measurement of permeability using MRI, which observes the penetration of contrast medium through the membrane. This method can be applied in both animal models and clinical measurements [43].

### Barrier experiments

**FIGURE 1:**
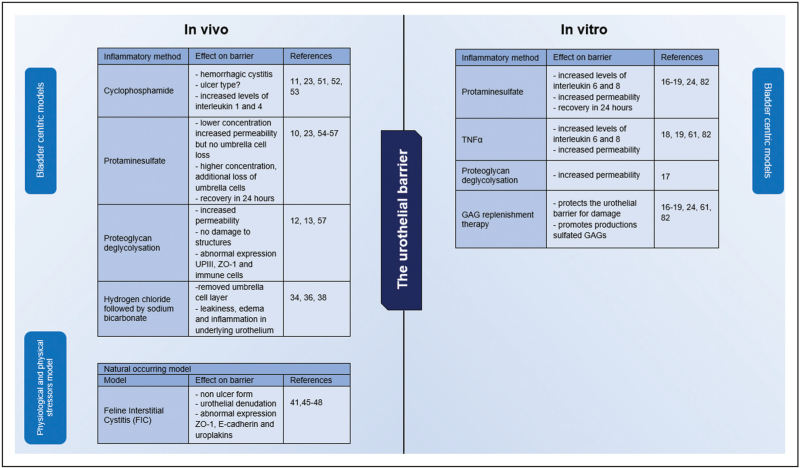
Overview of in vivo and in vitro barrier assessment in bladder urothelium.

Several decades ago, Lavelle *et al.* measured water and urea diffusion in the mammalian bladder, revealing low permeability in normal urothelium, which temporarily increased after inducing inflammation [17,35]. IC/BPS patients have an increased permeability, similar for patients treated with PS [44]. In vitro, instillations of PS in NPU increased permeability temporarily for 24 h [13]. HA treatment before damage with TNF-α protected the impermeability of the bladder wall [15]. A summary is given in Fig. 1.

## THERAPEUTIC CORRECTION OF BARRIER DYSFUNCTION

Loss of the GAG layer is a key factor in IC/BPS, hence commercial GAG products are available, for intravesical and oral use. Various intravesical GAG products are available, each with different registrations and usage worldwide. The oral product, pentosan polysulfate (∼7000 kDa), is the only oral medication approved by the U.S. Food and Drug Administration. A few percentage of the dose passes into the urine, where it is believed to have its efficacy [45].

The response rates and level of clinical efficacy vary, and there are several possibilities for the suboptimal response rates. First, the commercial GAGs are linear polymers that likely lie flat, whereas the natural PGs form a thicker, three-dimensional layer. In addition, none of these products have been optimized for continuous coverage of the bladder surface, allowing intermittent penetration of inflammatory and painful urinary solutes. To improve upon these characteristics, “SuperGAGs” have been developed, which are crosslinked GAG molecules with a higher molecular weight (million range). SuperGAGs forms a 3-dimensional structure, adhering tightly to the bladder surface and creating a thicker bound water layer. Studies demonstrated that they are more effective in restoring impermeability compared to normal GAGs [46,47].

## FUTURE RESEARCH PERSPECTIVES INTO MECHANISMS

This review examined the urothelial barrier in IC/BPS, through in vivo and in vitro studies. Numerous papers show loss of the barrier as an important pathophysiological characteristic. Most studies focus on specific disruption of the urothelial barrier, although some studies investigate inflammatory or neurogenic causes as well [48,49]. These models appear to be interconnected, as disruption of the apical layer triggers: inflammation, increased noxious neural signaling, increased bowel permeability, and loss of the barrier function. Bowel inflammation alone can induce bladder- inflammation and increased permeability through neuroinflammatory crosstalk, which might explain the common occurrence of IC/BPS and irritable bowel syndrome (IBS) [50]. IC/BPS shows clear signs that it is a systemic disease with heavy involvement of the immune system [51^▪^]. Transcriptome studies further reinforce this perspective, emphasizing the importance of inflammatory and immune pathways [52^▪^,^53^].

However, there is no definite explanation yet for the failure of the normally rapid recovery of the apical layer and normal impermeability in response to injury. Further research is needed to determine whether inflammation or permeability plays primary or secondary roles. In vitro studies, as well as in vivo studies, offer possibilities to identify the involvement of specific molecules in specific subtypes of IC/BPS. In addition to functionality, the influences on cellular level or secondary cascading effects that explain the full pallet of IC/BPS symptoms are given more consideration [9,15,38].

## CONCLUSION

In conclusion, various models to compromise the urothelial barrier have been conducted and have demonstrated similar characteristics to the histopathological findings in IC/BPS biopsies. In vitro studies, along with in vivo studies, provide opportunities to identify the involvement of specific molecules and pathologic pathways in IC/BPS that may help us better stratify this heterogenous group of patients. Further research is warranted to elucidate the primary or secondary role of permeability, together with inflammatory and neurogenic causes of the disease.

## Acknowledgements

*None.*

### Financial support and sponsorship

*None.*

### Conflicts of interest

*Robert E. Hurst has an advisory function at commercial company Glycologix Inc. Glycologix Inc. has had no part in organizing or facilitating this review.*

## REFERENCES AND RECOMMENDED READING

Papers of particular interest, published within the annual period of review, have been highlighted as:

▪ of special interest
▪▪ of outstanding interest
